# TRENDS AND RESEARCH FRONTIERS ON GUT MICROBIOTA AND STUNTING: A BIBLIOMETRIC INSIGHT FROM 2009 TO 2025

**DOI:** 10.1590/S0004-2803.24612025-142

**Published:** 2026-06-05

**Authors:** Rafli Z KAMIL, Nurul HASNIAH, Heni RIZQIATI, Linda WINDIARTI, Tyas UTAMI, Endang S RAHAYU

**Affiliations:** 1 Diponegoro University, Faculty of Animal and Agricultural Sciences, Food Technology Study Program, Semarang, Indonesia.; 2 College of Holistic Health Sciences, Department of Nutrition Science, Purwakarta, Indonesia.; 3 Gadjah Mada University, Faculty of Agricultural Technology, Department of Food and Agricultural Product Technology, Yogyakarta, Indonesia.

**Keywords:** Bibliometric analysis, gut microbiota, malnutrition, stunting, Análise bibliométrica, microbiota intestinal, desnutrição, atraso no crescimento

## Abstract

**Background:**

Stunting, a chronic consequence of undernutrition, continues to affect millions of children under five years of age worldwide, particularly in developing countries, with long-term impacts on physical growth, cognitive development, and economic productivity.

**Methods:**

This study provides a bibliometric analysis of 172 publications indexed in Scopus from 2009 to 2025, aiming to identify trends, key contributors, core sources, thematic evolution, and future research directions in this emerging field.

**Results:**

Results indicate a steady annual growth rate of 11.85%, with peak publication output in 2021, reflecting global attention. Influential contributions were led by Gordon JI, Barratt MJ, and Ahmed T, with the United States, France, Bangladesh, China, and Indonesia identified as the most active countries, and institutions such as Washington University and the International Centre for Diarrhoeal Disease Research (Bangladesh) playing pivotal roles. The most productive journals included PLOS ONE, Frontiers in Microbiology, Nutrients, and Gut Microbes, with Subramanian S serving as the foundational reference. Keyword analysis revealed a thematic shift from “malnutrition” and “stunting” toward mechanistic terms such as “dysbiosis,” “environmental enteric dysfunction,” and interventions including “probiotics” and “prebiotics.” Emerging themes highlight moderate acute malnutrition and microbiota-directed interventions as promising frontiers.

**Conclusion:**

This bibliometric analysis provides valuable insights into research trends, influential contributors, and emerging areas, offering a foundation for guiding future studies and supporting global strategies to address stunting as a critical public health challenge.

## HIGHLIGHTS

• A bibliometric study covering the years 2009 to 2025 examined 172 publications indexed in Scopus to chart research trends related to gut microbiota and stunting.

• The analysis utilized the Bibliometrix R-package and VOSviewer to pinpoint key authors, institutions, journals, and the evolution of themes.

• The research output demonstrated an annual growth rate of 11.85%, with significant contributions from the USA, France, Bangladesh, China, and Indonesia.

• Emerging themes highlight probiotics, metagenomics, and moderate acute malnutrition as promising areas for addressing childhood stunting in the future.

## RESUMO

**Contexto:**

O atraso no crescimento (stunting), uma consequência crônica da desnutrição, continua a afetar milhões de crianças menores de cinco anos em todo o mundo, particularmente em países em desenvolvimento, com impactos de longo prazo no crescimento físico, no desenvolvimento cognitivo e na produtividade econômica.

**Métodos:**

Este estudo apresenta uma análise bibliométrica de 172 publicações indexadas na Scopus no período de 2009 a 2025, com o objetivo de identificar tendências, principais contribuidores, fontes centrais, evolução temática e direções futuras de pesquisa neste campo emergente.

**Resultados:**

Os resultados indicam uma taxa de crescimento anual constante de 11,85%, com pico de publicações em 2021, refletindo a atenção global ao tema. Contribuições influentes foram lideradas por Gordon JI, Barratt MJ e Ahmed T, sendo Estados Unidos, França, Bangladesh, China e Indonésia os países mais ativos, e instituições como a Washington University e o International Centre for Diarrhoeal Disease Research (Bangladesh) desempenhando papéis fundamentais. As revistas mais produtivas incluíram PLOS ONE, Frontiers in Microbiology, Nutrients e Gut Microbes, tendo Subramanian S como referência fundamental. A análise de palavras-chave revelou uma mudança temática de “desnutrição” e “atraso no crescimento” para termos mecanísticos como “disbiose”, “disfunção entérica ambiental” e intervenções como “probióticos” e “prebióticos”. Temas emergentes destacam a desnutrição aguda moderada e intervenções direcionadas à microbiota como fronteiras promissoras.

**Conclusão:**

Esta análise bibliométrica fornece insights valiosos sobre tendências de pesquisa, contribuidores influentes e áreas emergentes, oferecendo uma base para orientar estudos futuros e apoiar estratégias globais para enfrentar o atraso no crescimento como um desafio crítico de saúde pública.

## INTRODUCTION

Malnutrition remains a major global health problem, involving both undernutrition, which is a deficiency of essential nutrients, and overnutrition, characterized by excessive nutrient intake. Undernutrition is especially common among children under five, particularly in developing countries. In children, this condition can be classified based on anthropometric measurements into stunting, wasting, and underweight, with stunting indicating a long-term issue. Furthermore, undernutrition can be categorized by severity into moderate acute malnutrition (MAM), where children face increased health risks but do not yet require medical treatment, and severe acute malnutrition (SAM), a more serious condition that can lead to death if not medically addressed^1^. If these acute conditions are not properly prevented or treated, children may develop chronic malnutrition, often resulting in stunting^2^. The negative effects of stunting extend beyond physical growth delays, including cognitive development problems that can lead to poorer educational outcomes and lower income in later life^3^ ^,^ ^4^. Therefore, stunting is a cycle that needs to be broken. Many studies have explored the causes of stunting in children, such as inadequate energy and nutrient intake (including maternal nutrition), recurrent infections, low maternal education, low household income, and poor socioeconomic conditions^5^ ^-^ ^7^. In recent research, there is also a growing focus on the connection between childhood stunting and the composition of the gut microbiota.

The gut microbiota is a dynamic community of bacteria, viruses, and fungi that plays a vital role in nutrient metabolism, immune function, and gastrointestinal health^8^. Predominantly, the gut microbiota is made up of anaerobic bacteria, with Firmicutes accounting for approximately 60%, and Bacteroidetes and Actinobacteria comprising about 10%, while the remaining 30% consists of other microbial species^9^. The balance of gut microbiota composition has gained significant attention from researchers, particularly concerning its association with various non-communicable diseases, including obesity, diabetes, coronary heart disease, colon cancer, and mental health disorders^10^ ^-^ ^15^ ^,^ and it is increasingly being linked to stunting. Dysbiosis, a condition marked by an overrepresentation of non-beneficial microflora such as *Enterobacteriaceae*, *Escherichia*, and *Shigella*, can negatively impact the host‘s physiological health^8^. This imbalance has been observed in stunted children, who tend to have non-beneficial microflora. Pathogenic and invasive microflora can cause infections in the digestive tract, disrupting nutrient absorption^16^. Several factors, including age, birth method, exclusive breastfeeding, diet, and antibiotic use, can lead to an imbalance in an individual‘s gut microbiota composition^8^. With ongoing research on gut microbiota, it is now considered a promising approach to uncovering the underlying causes of stunting in children, although this area of study is not as extensively researched as other non-communicable diseases.

With the increasing number of publications in this field, gaining a comprehensive understanding of the research landscape is imperative. Since this is a relatively new area of study, it is important to outline the trends, directions, and scientific contributions related to gut microbiota and stunting in children, which have not yet been systematically documented. This effort is vital to help researchers interested in the topic understand the field‘s development, identify significant contributing journals and authors for potential collaboration, and pinpoint areas needing further investigation. Consequently, a bibliometric analysis was carried out to map research topics related to gut microbiota and stunting in children, aiming to answer several relevant questions, such as the following:

1. What are the most pertinent journals and key reference articles?
2. Which documents, authors, institutions, and countries are the most significant contributors to research in this area?
3. What are the most commonly used keywords, and how has the thematic focus changed over time?
4. What are the current trends in scientific publications concerning gut microbiota and stunting in children?
5. What research gaps remain that could be explored to support future studies?

This study aims to systematically elucidate the intellectual framework and developmental trajectory of research concerning gut microbiota and stunting in children. It aims to identify areas that require further investigation and to suggest evidence-based recommendations for future research agendas. These agendas should align with efforts to address stunting through approaches focused on gut microbiota composition.

### Colonization of gut microbiota in the colon

The human digestive tract hosts a diverse array of microbial taxa, the balance of which can significantly influence the host‘s phenotype, particularly in terms of health status and the prevalence of non-communicable diseases. This microbial community is referred to as the gut microbiota. It is estimated that the number of microbes residing in the adult human digestive tract reaches 10^14^ cells, which is ten times greater than the number of human body cells^17^ ^,^ ^18^. Moreover, the diversity of gut microbiota composition is dynamic, beginning at birth and continuing into adulthood. The diverse composition of the gut microbiota can form a symbiotic relationship beneficial to human health, such as aiding in the synthesis of vitamins and proteins, stimulating the immune system, and facilitating the digestion and absorption of nutrients^19^ ^,^ ^20^. This is particularly true if the digestive tract is dominated by obligate anaerobic bacteria such as *Bifidobacterium*. Conversely, if the digestive tract is dominated by pathogenic microflora, these microbes can produce metabolites such as putrefactives and toxins that may cause infections and the onset of various diseases^21^ ^-^ ^23^. In general, bacteria, yeasts, and viruses in the digestive tract are collectively referred to as gut microbiota. Bacteria, as the dominant microflora, are composed of approximately 60% from the phylum Firmicutes, 10% from the phyla Bacteroidetes and Actinobacteria, and about 30% consists of other species^9^. However, these proportions are highly dynamic due to influences such as age and birth method, diet, environmental factors, antibiotic use, and the consumption of probiotics or prebiotics^8^ ^,^ ^24^.

The colonization of gut microbiota commences at birth and continues to evolve throughout an individual‘s lifespan^16^. In neonates, the initial microbiota predominantly comprises anaerobic bacterial groups, including Enterobacteria, Streptococci, and Enterococci. In contrast, the gastrointestinal tract of children is more densely populated by *Bifidobacterium* spp. and *Faecalibacterium* spp.^25^. During gestation, vertical transmission of gut microbiota from mother to fetus occurs^26^. However, gestational age and delivery method are critical determinants of gut microbiota colonization. Infants delivered via cesarean section exhibit reduced gut microbiota diversity compared to those born vaginally, with an increased prevalence of *Staphylococcus*. This is attributed to the absence of exposure to the vaginal microbiota during cesarean delivery, leading to heightened exposure to *Staphylococcus* microorganisms typically present on the skin^27^ ^-^ ^29^. On the other hand, full-term birth at nine months is associated with enhanced microbiota diversity and a higher abundance of *Bifidobacterium*, due to prolonged exposure to maternal gut microbiota^30^ ^,^ ^31^. Infants who are exclusively breastfed are also observed to possess a greater quantity of beneficial microflora, such as *Bifidobacterium*. This is because breast milk inherently contains prebiotics, specifically galacto-oligosaccharides, which are selectively utilized by non-pathogenic bacteria^32^. Consequently, breastfeeding can inhibit the proliferation of *Enterobacteriaceae* in infants, which is associated with malnutrition-enteropathy, a condition resulting from digestive tract disorders.

Alterations in the gut microbiota composition commence during the weaning phase when infants begin to consume foods complementary to breast milk. This transition is characterized by an increase in Firmicutes, which generally remains stable into adulthood. These modifications are further influenced by dietary habits and age^33^. The impact of dietary patterns on gut microbiota composition is categorized into the *Prevotella* enterotype (P-type) and the *Bifidobacterium/Bacteroides* enterotype (BB-type). The P-type is associated with a high carbohydrate intake, as observed in adults from Yogyakarta and Bali (Indonesia) and Khon Kaen (Thailand). Conversely, the BB-type is linked to the consumption of animal protein sources and saturated fats, as identified in adults from Bangkok (Thailand), Beijing and Lanzhou (China), Tokyo and Fukuoka (Japan), as well as Taipei and Taichung (Taiwan)^34^. The modernization of dietary patterns in large urban areas indicates that enterotypes may differ from those in smaller cities. Newborns in Indonesia initially exhibit a BB-type distinct from their mothers’ P-type^35^. This enterotype subsequently transitions to a P-type following weaning and the introduction of complementary foods rich in complex carbohydrates and fruits. Although most *Prevotella* species are commensal gut bacteria, certain species possess pathobiontic properties and have been implicated in opportunistic infections, such as inflammatory bowel disease and metabolic disorders^36^ ^,^ ^37^. The predominance of *Bifidobacterium/Bacteroides* in the digestive tract is considered advantageous, as *Bifidobacterium* can protect the gut environment and is relatively prevalent in healthy individuals^38^ ^-^ ^40^. This underscores the importance of maintaining a balanced diet, which is associated with the equilibrium of gut microbiota composition, known to influence the host‘s health condition.

### The relationship between gut microbiota composition and stunting

The digestive tract fulfills two primary functions. Firstly, it acts as the site for the breakdown of macromolecules and the absorption of nutrients necessary to satisfy energy requirements. Secondly, it functions as a defense mechanism against pathogenic microorganisms. Consequently, interactions may occur between the composition of the gut microbiota and the host‘s health and nutritional status. The association between undernutrition in children and the gut microbiota creates a detrimental cycle known as environmental enteric dysfunction (EED). EED is an acquired enteropathy of the small intestine, characterized by inflammation of the digestive tract, villous atrophy, and a reduced crypt-to-villus ratio^41^. EED has been associated with several adverse outcomes, including chronic malnutrition (stunting), wasting, and diminished vaccine efficacy in children residing in resource-limited environments. Five highly interdependent mechanisms may connect EED to poor health outcomes: 1. increased intestinal permeability leading to bacterial or antigen translocation, 2. chronic intestinal inflammation without translocation, 3. malabsorption, 4. hormonal disturbances, and 5. microbiome disruption^41^ ^,^ ^42^. Infections caused by enteric bacteria result in alterations in the gut microbiota composition, characterized by a low diversity of beneficial bacteria. This condition can damage the intestinal mucosa and impair intestinal permeability, leading to inflammation and nutrient absorption failures. Furthermore, infection of the intestinal epithelial layer can result in a compromised immune system. Histological changes in the intestine due to infection are highly probable, such as villous atrophy, mucosal thinning, crypt branching, and brush border narrowing^43^.

**FIGURE 1:**
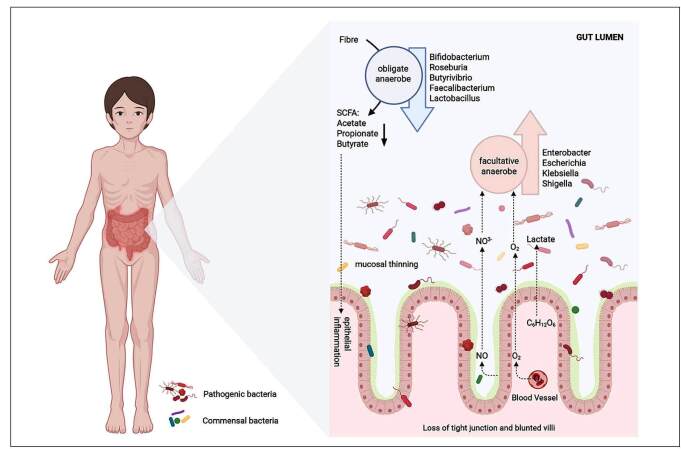
The cross-talk mechanism of gut microbiota and stunting in children. Created using biorender.com.

Numerous studies have indicated that malnutrition, particularly stunting, is associated with reduced diversity in gut microbiota composition, characterized by a predominance of several detrimental bacterial species, impaired intestinal function, and a decrease in the production of beneficial metabolites by commensal bacteria. Figure 1 illustrates the potential crosstalk mechanism between gut microbiota composition and stunting in children. Elevated levels of the Proteobacteria phylum have been observed in children experiencing stunting, alongside an increase in enteric pathogenic bacteria such as *Enterobacter, Escherichia, Klebsiella, and Shigella* ^44^ ^-^ ^50^. The high prevalence of these pathogenic bacteria correlates with decreased levels of beneficial bacteria, including *Bifidobacterium*, *Roseburia*, *Butyrivibrio*, *Faecalibacterium*, and *Lactobacillus*, which are obligate anaerobic enteric bacteria capable of colonizing the intestinal mucosa^43^ ^,^ ^50^. The causal relationship between microbiota differences and malnutrition remains ambiguous, as it is unclear whether microbiota alterations contribute to malnutrition or vice versa, given the cyclical nature of this relationship. However, the dominance of these unfavorable bacteria can lead to competition for colonization of the intestinal mucosa. Extensive colonization by pathogenic bacteria can result in infection and inflammation, potentially increasing intestinal permeability, a condition referred to as gut leakage^51^. Gut leakage renders the intestinal environment more aerobic than under normal conditions, thereby fostering the growth of pathogenic bacteria, which are typically facultative anaerobes. Infection and inflammation of the intestinal epithelium can also disrupt nutrient absorption due to shortened villi, thinning of the mucosa, branching of the crypts, and narrowing of the brush border^52^.

The association between microbiota dysbiosis and stunting may be attributed to the reduced energy supply for colonocyte cells within the intestine. Short-chain fatty acid (SCFA) metabolites are generated through the fermentation of indigestible polysaccharides by microbes such as *Bifidobacterium*, *Faecalibacterium*, *Akkermansia*, and *Lactobacillus* ^53^. SCFAs exhibit anti-inflammatory properties, lower the pH of the intestinal environment, and thereby create conditions that are unfavorable for pathogenic bacteria^54^ ^,^ ^55^. Acetate, propionate, and butyrate are the predominant SCFAs produced by gut microbiota^55^. SCFAs, particularly butyrate, can serve as an energy source for colonic epithelial cells (colonocytes). The production of CO_2_ by colonocytes indicates that these cells utilize SCFAs, especially butyrate, as an energy source through the β-oxidation process^56^. Conversely, acetate and propionate are transported to the liver via the hepatic portal vein, where they can be utilized as substrates for glucose production through gluconeogenesis^57^. It is established that SCFA levels are lower in stunting conditions compared to normal conditions^50^ ^,^ ^58^ ^,^ ^59^. Due to the limited amount of butyrate available for oxidation in colonocytes, glucose in the blood vessels of colonocyte cells is metabolized via anaerobic glycolysis, resulting in the production of lactate, which is subsequently released into the lumen^60^. This lactate production is accompanied by the generation of nitrate radicals (NO_3_^-^) that can induce infection and inflammation in the intestinal epithelium. Damage to the intestinal epithelial layer can subsequently alter the oxygen gradient in the lumen, rendering it more aerobic. Additionally, NO_3_^-^ released into the lumen can also serve as an electron acceptor that supports the growth of facultative anaerobic microorganisms^60^.

## METHODS

Bibliometric analysis can be performed utilizing various databases, including Scopus, Clarivate Analytics‘ Web of Science, and Dimensions^61^. These databases facilitate the acquisition of a systematic and comprehensive overview of a specific topic, identifying research clusters supporting the topic, and visualizing key concepts and their interrelationships. This particular bibliometric analysis will employ the Scopus database due to its broader coverage in the fields of medicine, clinical research, and nursing^62^. Scopus encompasses approximately 82% more journals than Web of Science, although it still falls short of Dimensions, which boasts the most extensive coverage^63^.

Boolean operators will be employed to enhance and refine the keyword search, thereby developing a more comprehensive and precise keyword search strategy pertinent to the topic of gut microbiota and stunting. The Boolean operator “AND” will be utilized to integrate key concepts, ensuring that the selected keywords encompass all critical terms. Conversely, the “OR” operator will be employed to broaden the search scope for overlapping terms. Quotation marks, or the exact phrase operator (“), will be used to locate exact phrases in the specified order, while the truncation symbol or wildcard (*) will replace one or more characters at the end of a word to search for various word forms. The grouping symbol or nesting (()) will be used to organize operators to control the search sequence. This methodology facilitates the inclusion of pertinent studies, including those that may primarily focus on specific components but also offer significant insights within the broader context of gut microbiota and stunting. Based on this, the keyword search strategy to be applied in the bibliometric analysis is as follows:

(“gut microbiota” OR “intestinal microbiota”) AND (child OR infant OR toddler OR pediatric) AND (stunting OR “growth failure” OR malnutrition OR undernutrition)

**FIGURE 2:**
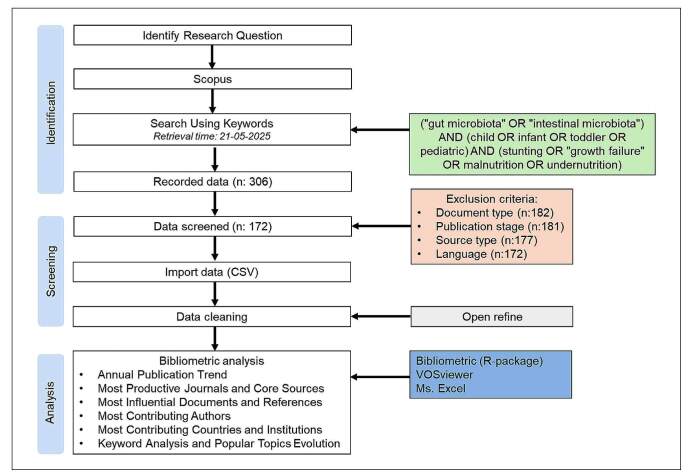
Workflow of bibliometric analysis on gut microbiota and stunting children.

The bibliometric analysis conducted included inclusion and exclusion criteria to ensure that only relevant articles were considered. The inclusion and exclusion criteria can be seen in the Table 1. Metadata were collected in accordance with the established search strategy and the application of inclusion and exclusion criteria, subsequently exported in CSV format for further analysis. The CSV files will be analyzed using R Studio (v. 4.4.2), utilizing the R package Bibliometrix (v. 5.0.0), and VOSviewer application (v. 1.6.20). The workflow of analysis can be seen in Figure 2.

**TABLE 1:** Inclusion and exclusion criteria used in this bibliometric analysis.

Criteria	Inclusion	Exclusion
Language	English	Other languages
Year of publication	2009-2025	Before 2009
Document type	Articles	other
Source type	Journal	other
Publication stage	Final	Article in press

## RESULTS

### Database characteristics

Table 2 presents a comprehensive overview of the database utilized, sourced from Scopus, following specific inclusion and exclusion criteria. The keyword search strategy revealed that the earliest articles addressing the topic of gut microbiota and stunting were published in 2009. From 2009 to 2025, a total of 172 documents were published across 102 sources. The subject under investigation exhibits an annual growth rate of 11.85%, signifying a notable upward trend in scientific publications and indicating sustained global research interest. It is crucial to recognize that while a high growth rate reflects a dynamic and expanding field, it must be accompanied by the quality of publications to ensure meaningful scientific advancement. A balance between growth and quality is imperative, as unchecked rapid growth may adversely affect the journal’s impact factor^64^. The document‘s average Age of 5.32 years suggests a balance between older and newer publications, indicating a robust research foundation and ongoing scholarly interest. The average citation per document of 50.81 underscores the substantial influence of each publication within the scientific literature. This metric highlights the topic‘s strong relevance and contribution to scientific progress, as well as the potential presence of frequently cited key articles^65^. A total of 8,825 references were employed in these 172 documents. There were 2,179 keywords plus (ID) and 424 authors‘ keywords utilized throughout all documents. Within the examined topic, there were 1,363 authors, with 2 authors contributing to single-authored documents. The average number of authors per document was 10.7, indicating a high degree of collaboration within this research community. Furthermore, 58.14% of the documents resulted from international collaboration, signifying the global interest in this topic and the involvement of networks of researchers across countries.

**TABLE 2:** Main information of collected publications relating to gut microbiota and stunting in children.

Description	Results
**Main information about data**	
timespan	2009:2025
Sources (Journals, Books, etc)	102
Documents	172
Annual Growth Rate %	11.85
Document Average Age	5.35
Average Citations per Document	50.81
References	8825
**Document contents**	
keywords Plus (ID)	2179
Author’s Keywords (DE)	424
**Authors**	
authors	1363
Authors of Single-Authored Documents	2
**Authors collaboration**	
single-authored Documents	2
Co-Authors per Document	10.7
International Co-authorships %	58.14
**Document types**	
article	172

### Annual publication trend

**FIGURE 3:**
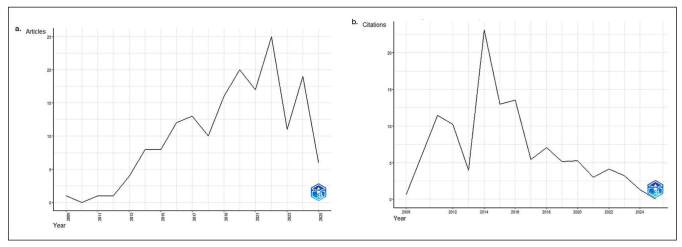
(A) annual trend in the number of publications related to gut microbiota and stunting in children. (B) the change in annual citations per year.

Figure 3A depicts an upward trajectory in the number of publications commencing in 2009, reaching a peak in 2021 with approximately 25 articles. Initially, in 2009, a solitary article addressed the topic of gut microbiota and stunting in children, and this trend remained relatively stable, with only one article published annually until 2013, except in 2010. Interest in this subject began to intensify in 2014, as evidenced by the publication of four articles that year. This increase reflects a growing research interest in gut microbiota and stunting. Subsequently, fluctuations occurred, including a notable decline in 2022 and 2024, although 2023 experienced a temporary increase. The decline in articles post-2021 is attributed to a shift in research and publication priorities within the health sector towards the Covid-19 pandemic. The pronounced decrease in 2025 may be due to the year being incomplete or the data not being fully collected. Overall, this trend demonstrates positive growth with an annual rate of 11.85%, indicating that this topic continues to engage the scientific community‘s interest. In addition, Figure 3B depicting average citations per year indicates that the highest average citations per article were recorded in 2014, surpassing 20 citations per article. Following this peak, a notable decline in citation numbers is observed, persisting through 2025. This downward trend may be attributed to the emergence of new publications that have not yet garnered widespread citation or a shift in research priorities. Despite the increasing volume of publications, the graph underscores the necessity of maintaining scientific quality and impact to ensure continued relevance within the academic community.

### Most productive journals and core sources 

**FIGURE 4:**
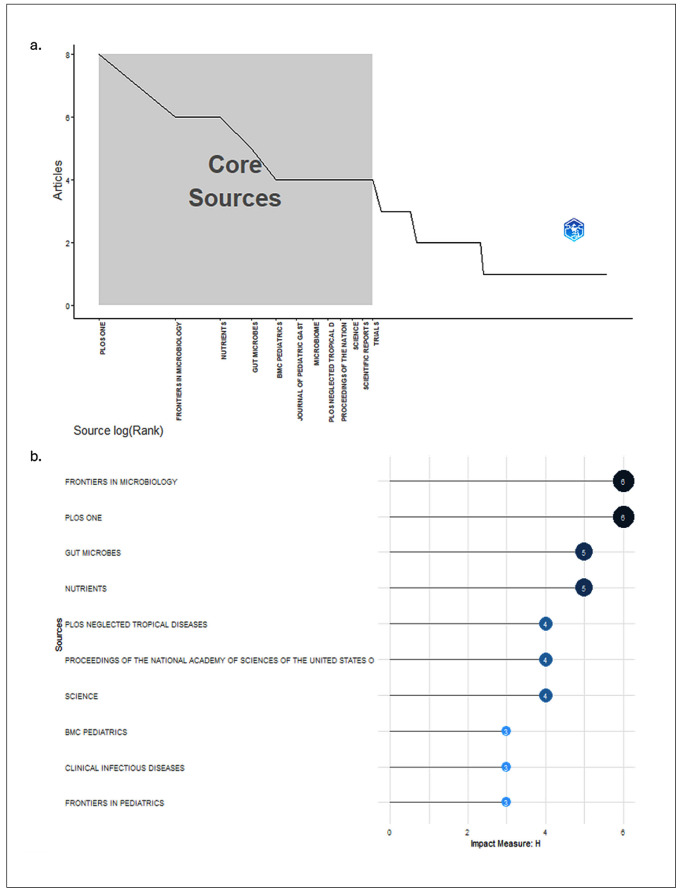
(A) core sources by bradford’s law. (B) source’s local impact by h-index.

Bradford’s Law, a fundamental principle in bibliometrics, elucidates the uneven distribution of scientific articles across journals^66^. According to this principle, a limited number of core journals encompass the majority of pertinent articles within a specific field, while the remaining articles are dispersed across a larger array of less productive journals. Bradford’s law is instrumental in identifying core journals within a specific research domain, thereby facilitating efficient management of information resources^67^. Figure 4A illustrates that the majority of articles concerning the topic of gut microbiota and stunting in children are concentrated in several key journals, specifically PLOS ONE, Frontiers in Microbiology, Nutrients, and Gut Microbes. These journals serve as pivotal “core sources” and are the most prolific in the study of gut microbiota and stunting in children. This is corroborated by the local impact of these sources, evaluated through the H-index, which reflects a journal’s contribution and influence within a particular research field. As depicted in Figure 4B, the journals PLOS ONE and Frontiers in Microbiology each possess an H-index of 6, while Gut Microbes and Nutrients each have an H-index of 5. This underscores the status of these journals as the most influential sources, frequently cited in related research, and as primary hubs for the dissemination of research findings in this field.

### Most influential documents and references

To identify the most impactful documents and references, one can look at those that receive the highest number of citations both on a global and local scale. Documents that are widely cited globally attract numerous references from a diverse range of publications across different fields, highlighting their significant influence and standing within the scientific community. Conversely, locally cited documents are those frequently referenced within a specific dataset under analysis, highlighting their significance and contribution to a particular research area^68^. The primary distinction between the two lies in the scope of citation: global citations pertain to the entire scientific literature, whereas local citations are confined to a specific research context. Both perspectives are crucial for comprehending influential literature from both broad and narrow viewpoints.

Table 3 lists the 15 documents with the highest global citation counts related the topic of gut microbiota and stunting in children. The article by Subramanian S ranked first, with 962 global citations, underscoring its significance as a primary reference. This is succeeded by Blanton LV and Wong J, with 580 and 521 citations, respectively, along with other influential articles such as Schwarzer M and Voreades N. The majority of these articles were published between 2011 and 2016 in highly esteemed journals, marking a pivotal period in the advancement of research in this domain. Within the locally analyzed dataset (Table 4), the article by Subramanian S also occupies the top position with 74 local citations, signifying its relevance in the context of this specific study. The articles by Schwarzer M and Chen RY followed by 17 and 8 local citations, respectively. The majority of the other documents exhibit low local citation counts, indicating that only a select few publications serve as core references. These findings are crucial for identifying key literature that can form the foundation for further studies on related topics.

**TABLE 3:** Top 15 most global cited documents.

Author, Year	Journal	DOI	Total Citations
Subramanian S, 2014	Nature	10.1038/nature13421	962
Blanton LV, 2016	Science	10.1126/science.aad3311	580
Wong J, 2014	American Journal of Nephrology	10.1159/000360010	521
Schwarzer M, 2016	Science	10.1126/science.aad8588	469
Voreades N, 2014	Frontiers in Microbiology	10.3389/fmicb.2014.00494	385
Kau AL, 2015	Science Translational Medicine	10.1126/scitranslmed.aaa4877	291
Reyes A, 2015	Proceedings of the National Academy of Sciences	10.1073/pnas.1514285112	232
Crane RJ, 2015	Food and Nutrition Bulletin	10.1177/15648265150361S113	193
Owino V, 2016	Pediatrics	10.1542/peds.2016-0641	189
Monira S, 2011	Frontiers in Microbiology	10.3389/fmicb.2011.00228	172
Brown EM, 2015	Nature Communications	10.1038/ncomms8806	168
Harper KM, 2018	PLOS Neglected Tropical Diseases	10.1371/journal.pntd.0006205	160
Raman AS, 2019	Science	10.1126/science.aau4735	154
Ghosh TS, 2014	PLoS ONE	10.1371/journal.pone.0095547	151

**TABLE 4:** Top 15 most local cited documents.

Author, Year	Journal	DOI	Local Citations	Global Citations
Subramanian S, 2014	Nature	10.1038/nature13421	74	962
Schwarzer M, 2016	Science	10.1126/science.aad8588	17	469
Chen RY, 2020	New England Journal of Medicine	10.1056/NEJMoa1916004	8	119
Kumar M., 2018	Metabolic Engineering	10.1016/j.ymben.2018.07.018	6	68
Grześkowiak L, 2012	Journal of Pediatric Gastroenterology and Nutrition	10.1097/MPG.0b013e318249039c	4	143
Kortekangas E, 2020	Paediatric and Perinatal Epidemiology	10.1111/ppe.12623	3	14
Balasubramaniam C, 2021	European Journal of Nutrition	10.1007/s00394-021-02571-7	3	8
Cowardin CA, 2019	Proceedings of the National Academy of Sciences	10.1073/pnas.1821770116	3	68
Masrul M, 2020	Open Access Macedonian Journal of Medical Sciences	10.3889/oamjms.2020.4209	2	8
Schwarzer M, 2023	Science	10.1126/science.ade9767	2	57
Kamil RZ, 2021	Microorganisms	10.3390/microorganisms9010127	2	28
Monira S, 2009	Journal of Pediatric Gastroenterology and Nutrition	10.1097/MPG.0b013e3181831867	2	11
Mayneris-Perxachs J, 2016	The American Journal of Clinical Nutrition	10.3945/ajcn.116.131797	2	84
Chang H-W, 2024	Nature Microbiology	10.1038/s41564-024-01628-7	1	21
Ordiz MI, 2017	The American Society of Tropical Medicine and Hygiene	10.4269/ajtmh.16-0617	1	39

**FIGURE 5:**
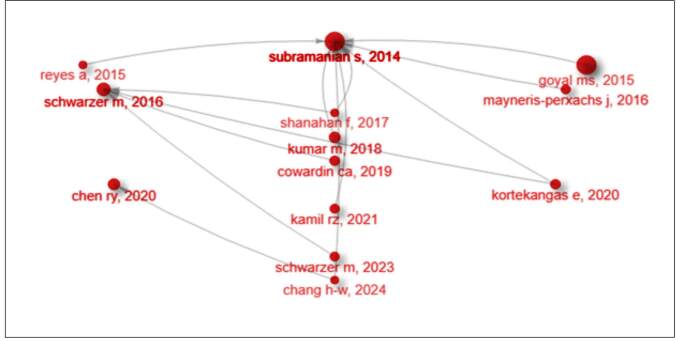
Main citation network historiography in the field of gut microbiota and stunting research.

The results of the bibliometric analysis, as visualized through the historiograph (Figure 5), indicate that the article by Subramanian S, is the most influential publication concerning the topic of gut microbiota and stunting in children. This article functions as a central node within the network due to its frequent citation by other studies, underscoring its pivotal role as a foundational scientific work in this domain. Several other works, such as those by Cowardin CA, Goyal MS, Kumar M, and Schwarzer M seem to create a research cluster that is closely linked to the primary article. This web of connections illustrates a seamless flow of knowledge from initial studies to later research efforts. Additionally, the inclusion of more recent works, like those by Chang H-W, Kamil RZ, and Schwarzer M, indicates that this subject continues to develop and attract researchers‘ interest over the last ten years. This network also implies the possibility of strong collaboration or intellectual influence among researchers, especially those who frequently appear at various points within the network.

### Most contributing authors

**FIGURE 6:**
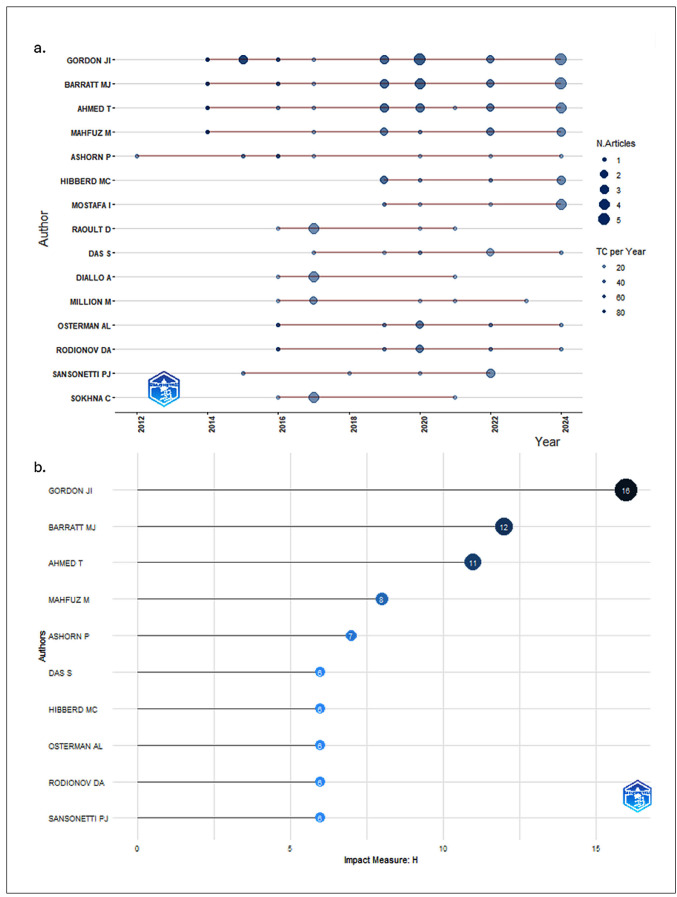
(A) author’s production over time. (B) authors’ local impact by H-indeks.

Data analysis from 2009 to 2025 highlights several key authors who have significantly advanced the understanding of gut microbiota and stunting. Figure 6A from the bibliometric analysis shows that certain authors have consistently offered valuable insights over the last ten years in the study of gut microbiota and stunting in children. Notably, Gordon JI, Barratt MJ, and Ahmed T have demonstrated high productivity and considerable scientific impact, as reflected in their numerous publications and high annual citation counts, especially between 2016 and 2020. They have published 21, 17, and 16 articles, respectively, with total citations reaching 2872, 2204, and 1760. Mahfuz M has also maintained a steady contribution since 2015, with a noticeable increase in publication activity from 2018 to 2021. In contrast, Ashorn P has been active since 2012, but his publication rate is more sporadic and less intense compared to the others. Authors like Hibberd MC, Mostafa I, and Raoult D began contributing in 2017, though in smaller numbers. Meanwhile, Das S and Diallo A were active during 2016 - 2017 but have not continued publishing in the following years.

Based on the Figure 6B illustrates the authors with the most global citations in the area of gut microbiota and child stunting, Gordon JI emerges as the leading figure, boasting 16 global citations. This underscores his prominence as a top expert in this field, both in terms of academic contributions and scientific impact. Trailing him are Barratt MJ and Ahmed T, with 12 and 11 global citations, respectively, highlighting the frequent citation of their work in scientific discourse. Mahfuz M and Ashorn P have garnered eight and seven global citations, reflecting their input to the field, though not as extensively as the top three authors. Meanwhile, authors like Das S, Hibberd MC, Osterman AL, Rodionov DA, and Sansonetti PJ each have six global citations. Despite having fewer citations, their research significantly advances the understanding of the link between gut microbiota and stunting. Overall, the graph showcases several researchers with considerable influence in this subject, serving as essential references and potential collaborators for future studies. In summary, a group of key authors, including Gordon JI, Barratt MJ, Ahmed T, and Mahfuz M, have shown strong research continuity and substantial scientific impact in the field of gut microbiota and stunting in children, playing a crucial role in the increase of articles since 2014 and acting as valuable reference points.

### Most contributing countries and institutions 

**FIGURE 7:**
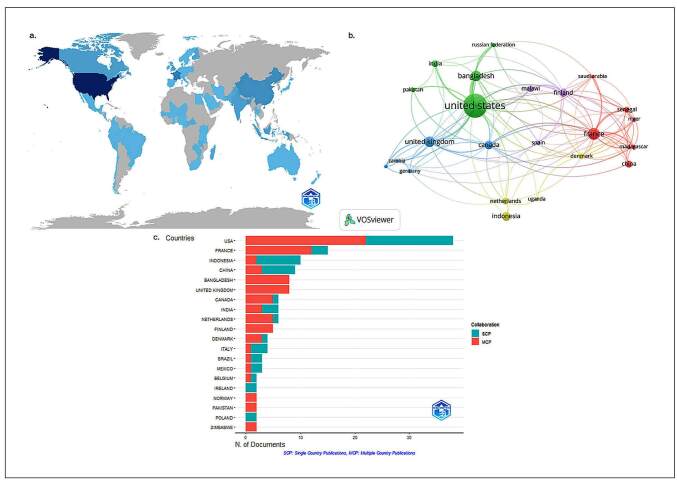
(A) country scientific contribution. (B) network of global research collaborations created by VOSViewer. (C) corresponding author’s country, scp: single country publications; mcp: multiple country publications.

Figure 7A and Table 5 present the frequency distribution of articles concerning the topic of gut microbiota and stunting by country, spanning the years 2009-2025. Although stunting is predominantly observed in developing nations, it is noteworthy that the majority of articles on gut microbiota and stunting originate from developed countries, including the USA, France, the UK, and Canada. This suggests that the subject of gut microbiota and stunting has garnered global interest. Additionally, countries such as China, Bangladesh, Indonesia, and India, where the prevalence of stunting is notably high, also make significant contributions to the advancement of research in this area. This indicates that research collaborations between technologically advanced countries and those with a high incidence of stunting are highly feasible for examining and addressing the issue, as can also be seen in Figure 7B-C . 

**TABLE 5:** Top 15 most contributing countries.

Country	Articles
USA	472
France	155
China	111
Bangladesh	102
UK	67
Canada	66
Indonesia	66
India	65
Finland	44
Madagascar	39
Pakistan	37
Malawi	36
Denmark	32
Senegal	27
Netherlands	26

This bibliometric visualization by VOSviewer (Figure 7B) illustrates the network of global research collaborations, with the United States prominently positioned as the central hub, as indicated by the largest circle and numerous connections. Other countries, such as France, the United Kingdom, Bangladesh, and Canada, also occupy significant roles. Colors represent collaborative clusters; for example, the United States forms a cluster with Bangladesh, India, and Pakistan, while France collaborates closely with African nations, including Senegal and Madagascar. The United Kingdom establishes its own cluster with Zambia and Germany, whereas Indonesia is grouped with the Netherlands, Uganda, and Denmark. The thickness of the lines signifies the strength of collaboration, with developed countries predominantly influencing the network, while developing nations, including Indonesia, participate but with still limited engagement.

According to the corresponding author‘s countries graph (Figure 7C), the United States emerges as the leading nation in terms of publication contributions on the subject of gut microbiota and stunting in children. A significant portion of these publications results from international collaborations or multiple country publications (MCP), underscoring the extensive involvement of American researchers in global research efforts. France, Indonesia, and China follow, each contributing a substantial number of publications. Notably, Indonesia and China exhibit a high degree of international collaboration (MCP), reflecting their active participation in cross-national research networks. In contrast, countries such as Bangladesh and the United Kingdom predominantly contribute through domestic publications or single country publications (SCP), highlighting the robustness of their internal research on this topic. Additionally, nations like Canada, India, the Netherlands, Finland, and Denmark make significant contributions through both national and collaborative publications. Meanwhile, countries such as Mexico, Italy, Brazil, Belgium, and Ireland, although contributing to a lesser extent, play a crucial role in enhancing the geographical diversity of the research landscape.

Table 6 presents the institutions that have significantly contributed to the advancement of research on gut microbiota and stunting. Notably, Washington University, the University of Virginia, the University of California, and The Ohio State University are the most active institutions, establishing the United States as the leading country in this field. Conversely, in France, the most active institutions include Institut Pasteur, Aix Marseille University, and Université De Lyon. In the context of developing countries, the International Centre for Diarrhoeal Disease Research in Bangladesh, Huazhong Agricultural University in China, Universitas Gadjah Mada in Indonesia and Mahidol University in Thailand are pivotal in advancing research on gut microbiota and stunting. Furthermore, the International Centre for Diarrhoeal Disease Research is identified as the most active institution within the Asian region.

**TABLE 6:** Top 15 most contributing affiliation.

Affiliation	Country	Articles
Washington University	USA	158
International Centre for Diarrhoeal Disease Research	Bangladesh	64
Institut Pasteur	France	38
Aix Marseille Univ	France	32
University of Copenhagen	Denmark	29
University of Virginia	USA	26
Institut Pasteur De Madagascar	Madagascar	23
University of British Columbia	Canada	23
University of California	USA	20
Aga Khan University Hospital	Pakistan	16
Huazhong Agricultural University	China	16
Universitas Gadjah Mada	Indonesia	16
Université De Lyon	France	16
Mahidol University	Thailand	15
The Ohio State University	USA	13

### Keywords analysis and popular topics evolution

**FIGURE 8:**
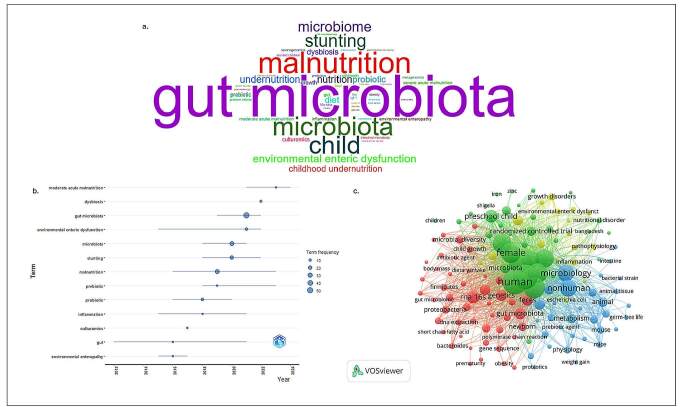
(A) word cloud produced from author’s keywords. (B) visualization map of trend topics analysis. (C) keywords co-occurrence analysis by VOSviewer.

Keywords encapsulate the essence of an article‘s message, thereby serving as a tool for examining topics and trends within a specific field of study. Keyword association analysis enables the rapid identification of the primary research focus within a given domain. Figure 8A presents a word cloud illustrating the frequency of 50 keywords employed by authors in the article database. The five most frequently used keywords are gut microbiota (53), malnutrition (27), microbiota (24), child (23), and stunting (16). Additionally, the term EED appears 10 times. This suggests that the subject of gut microbiota and stunting in children is associated with the phenomenon of EED.

The trend topics graphic (Figure 8B) illustrates the progression of key terms in research concerning gut microbiota and stunting in children from 2012 to 2024. The terms “malnutrition”, “stunting” and “mo­derate acute malnutrition”, which pertain to specific studies on undernutrition issues, have consistently emerged since 2016-2017, reflecting a focus on nutritional status in child growth. Concurrently, the terms “EED” and “environmental enteropathy” began to appear, suggesting a correlation between chronic gut disorders and instances of malnutrition and stunting. Conversely, “dysbiosis,” “microbiota,” and “gut microbiota” have consistently appeared since 2018, indicating a shift towards a mechanistic understanding of microbiota imbalances in ecosystems such as the gut. The convergence of these topics signifies an evolution in the study of malnutrition from the perspective of gut microbiota composition. Additionally, the terms “probiotics” and “prebiotics”, which started gaining attention in 2017, indicate a trend towards employing microbe-based interventions to enhance gut and nutritional health.

Figure 8C presents the co-occurrence of keywords as visualized through VOSviewer. The size and positioning of the terms denote their frequency and proximity in the literature. Keywords such as human, female, microbiology, preschool child, and feces are prominent, indicating a primary research focus on human studies, particularly concerning children and women. The color coding of each group signifies distinct thematic clusters. The green cluster pertains to clinical research, including controlled trials involving children. The red cluster emphasizes molecular aspects and microbiota composition, incorporating methodologies such as 16s RNA analysis. The blue cluster is associated with animal-based studies, predominantly utilizing mouse models to explore microbiota function in greater detail. Conversely, the yellow cluster underscores topics related to nutrition, inflammation, and growth disorders. The lines connecting keywords represent the thematic proximity within individual publications. Collectively, this map demonstrates that research on gut microbiota and stunting is multidisciplinary and interconnected, integrating clinical, molecular, and experimental approaches to elucidate the impact of microbiota on health, growth, and nutritional status, particularly in children.

**FIGURE 9:**
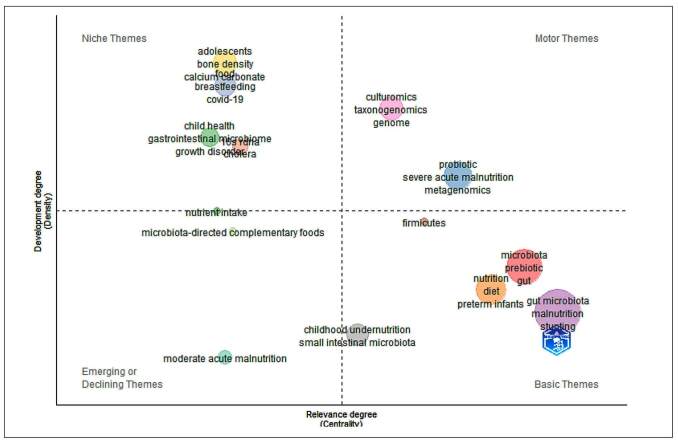
Thematic map of research on gut microbiota and malnutrition. Niche themes (upper left), motor themes (upper right), emerging or declining themes (lower left), and basic themes (lower right).

In employing co-occurrence analysis to elucidate relationships among keywords, a thematic map is essential for evaluating the impact and significance of a research theme (Figure 9). Utilizing the author‘s keywords, basic themes such as gut microbiota, malnutrition, and stunting can be further developed by investigating themes classified as motor themes, including probiotic, metagenomic, and culturomics. These topics are identified as active and rapidly evolving, suggesting that research on gut microbiota, malnutrition, and stunting can be advanced through molecular technology. Additionally, the lower left quadrant of the thematic map indicates emerging or declining themes, highlighting topics that are either in decline or have not yet been extensively developed in relation to the basic themes. This includes topics such as microbiota-directed complementary food, nutrient intake, and moderate acute malnutrition. According to the trend topics graph, moderate acute malnutrition has emerged as a novel topic of increasing scholarly interest. This trend suggests a shift in research focus towards the MAM category, a form of moderate malnutrition that can be managed at the community level without severe medical complications. Consequently, research trends are anticipated to pivot towards preventing the exacerbation of malnutrition cases within communities, rather than addressing chronic malnutrition or stunting. Conversely, niche themes relate to topics that lack a strong connection with the foundational themes.

## CONCLUSION

This bibliometric analysis provides a comprehensive overview of research on gut microbiota and stunting in children between 2009 and 2025, highlighting steady growth in publications and the emergence of a multidisciplinary field. Influential contributions from leading authors, institutions, and countries underscore the central role of collaborative research in advancing this domain, particularly between high-capacity institutions (e.g., Washington University, Institut Pasteur) and high-burden regions (e.g., Bangladesh, Indonesia). Key authors such as Gordon JI, Barratt MJ, and Ahmed T, alongside countries including the USA, France, China, and Bangladesh, have shaped the knowledge landscape. Core journals (PLOS ONE, Frontiers in Microbiology, Nutrients, Gut Microbes) and seminal works such as Subramanian S continue to anchor the field.

Current research trends emphasize the integration of clinical, molecular, animal-based, and nutritional approaches, with increasing attention to the gut microbiota’s role in malnutrition, environmental enteric dysfunction, and stunting. Thematic mapping further points that probiotics and metagenomics are poised to drive future advancements in this field, while MAM emerges as a promising new focus area. Strengthening international collaboration, advancing mechanistic insights into the microbiota and stunting relationship, and translating research findings into effective, scalable interventions remain critical priorities to address stunting as a pressing global health challenge.

## Data Availability

Data-available-upon-request
